# Long-term phosphorus fertilization reveals the phosphorus limitation shaping the soil micro-food web stability in the Loess Plateau

**DOI:** 10.3389/fmicb.2023.1256269

**Published:** 2024-01-11

**Authors:** Liangliang Li, Zhuzhu Luo, Lingling Li, Yining Niu, Yaoquan Zhang, Renyuan He, Jiahe Liu, Lili Nian

**Affiliations:** ^1^College of Resources and Environmental Sciences, Gansu Agricultural University, Lanzhou, China; ^2^State Key Laboratory of Arid Habitat Crop Science, Lanzhou, China; ^3^College of Forestry, Gansu Agricultural University, Lanzhou, China

**Keywords:** Loess Plateau, soil micro-food web, nematode metabolic footprint, decomposition pathway, phosphorus

## Abstract

The intricate decomposition pathways within soil micro-food webs are vital for cycling soil organic carbon and nutrients, influencing the quality, productivity, and sustainability of soil systems. However, the impact of diverse phosphorus addition on these organic decomposition pathways still needs to be explored. In an 8-year experiment, phosphorus (P) fertilizer was added at varying levels (0 kg ha^−1^, CK; 60 kg ha^−1^, P60; 120 kg ha^−1^, P120; and 180 kg ha^−1^, P180), to investigate the response of the soil micro-food web. The results revealed a significant effect of phosphorus addition on soil microorganisms and nematodes, with P60 exerting a greater influence than other treatments. At P60, the Shannon index of nematodes and fungi surpassed other treatments, indicating higher diversity, while the Shannon index of bacteria was lower. The Chao1 index of bacteria and fungi at P60 was higher, contrasting with the lower index for nematodes. Metabolic footprints of bacterivores and omnivores–predators (BFMF and OPMF) were higher at P60, while metabolic footprints of fungivores and plant parasites (FFMF and PPMF) were lower, signifying altered energy flow. Functional metabolic footprints and energy flow analysis unveiled a stable soil micro-food web structure at P60, with enhanced energy conversion efficiency. Network analysis illustrated positive correlations between fungi, fungivorous nematodes (FF), and omnivorous-predatory nematodes (OP) at P60, while P120 and P180 showed positive correlations among bacteria, bacterivorous nematodes (BF), and OP. Path analysis underscored the higher contribution rate of BF-C, FF-C, and OP-C to soil organic carbon at P60 compared with P120 and P180. These findings suggest that nutrient interactions between fungi and nematodes regulate soil micro-food web decomposition under low phosphorus concentrations. In contrast, interactions between bacteria and nematodes dominate at high phosphorus concentrations. The study indicates that adding phosphorus has nuanced bottom-up effects, intricately shaping the structure and activity of the pathways and underscoring the need for a comprehensive understanding of nutrient dynamics in soil ecosystems.

## 1 Introduction

**Figure 1:**
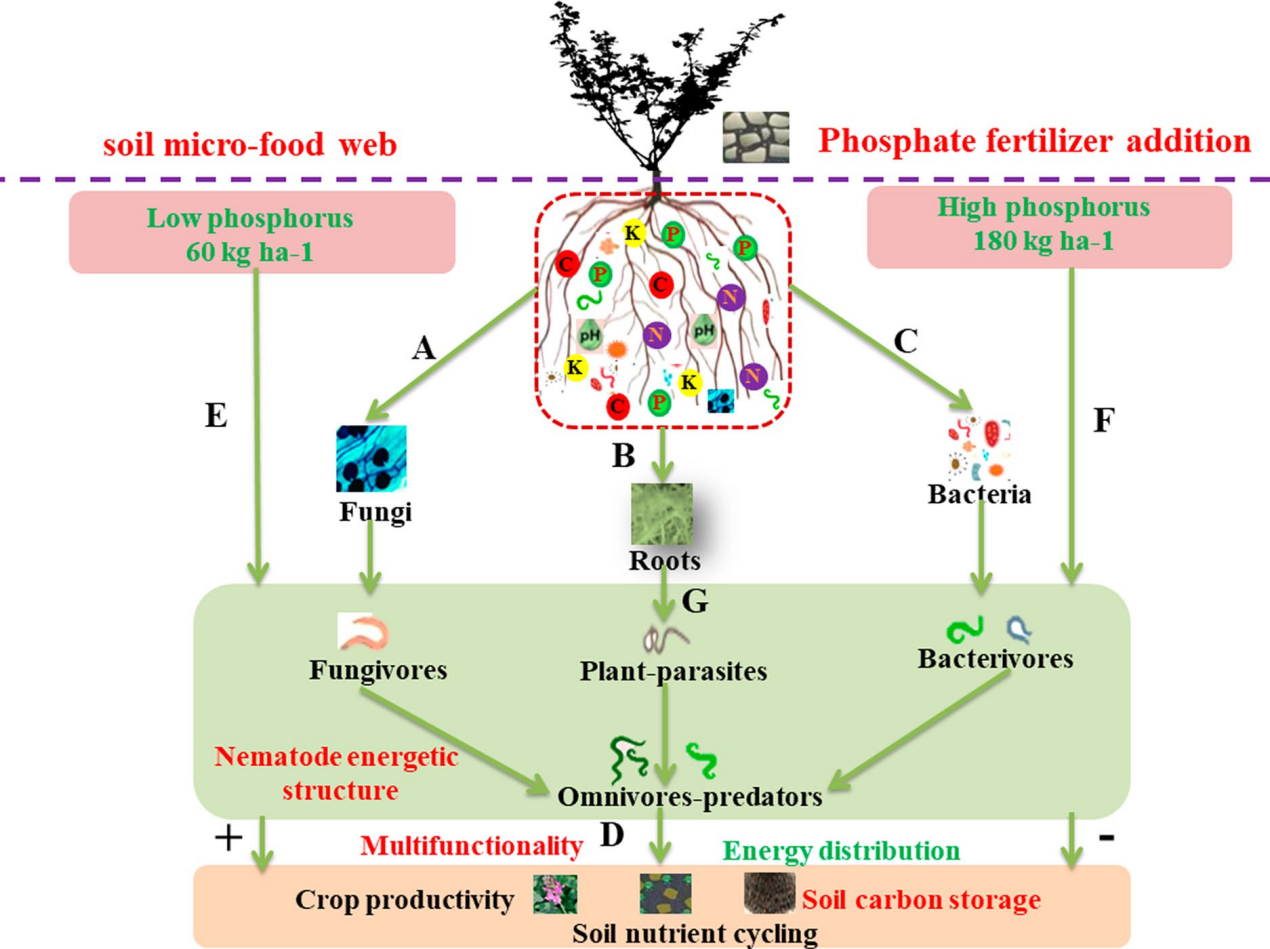
A conceptual framework showing how soil multifunctionality in sustainable agriculture emerges as the result of the underlying food web structure, which is fueled by energy and nutrients coming from phosphorus (P) fertilizer applications to the agroecosystem. Phosphorus fertilizers increase the energy flux through microbivores and herbivores by promoting soil microbial biomass **(A,C)** and plant growth **(B)**, respectively. These changes would increase the energy flux through omnivores-carnivores and alter nematode energetic structure, enhancing soil multifunctionality **(D)**. When nutrients were limited, plant root growth and rhizodeposition would be stimulated, and more energy flux through herbivores would disrupt the energy flux balance and change soil multifunctionality **(G)**. Conversely, when nutrients were sufficient, more energy flux by microbivores would favor soil multifunctionality **(E,F)**.

Soil is the largest organic carbon pool in terrestrial ecosystems, the carbon storage of which exceeds the sum of vegetation and atmospheric carbon pools (Lehmann and Kleber, 2015). Indeed, soil organic carbon (SOC) is crucial in providing ecosystem services and regulating the global carbon cycle. However, small changes in the global SOC pool will significantly impact atmospheric CO_2_ concentration (Kirschbaum, 2000). This change in SOC depends on the balance between plant inputs and microbial decomposition outputs, which can be further affected by soil phosphorus (P) fertilizers (Jackson et al., 2017). Mismanagement or misunderstanding of phosphorus fertilizers can have disastrous effects on soil quality and productivity (Poirier et al., 2014). An important agricultural management practice also provides carbon and nutrient sources to soil micro-food web through decomposition pathways (Das et al., 2012). The soil micro-food web represents a consumer–resource interaction network composed of “microbes–protozoa–nematodes” or “microbes–nematodes” and other soil microorganism groups with different functions (Lavelle, 1997). Modifications in the structure of these webs not only signify alterations in the energy pathways of organic matter but also exert a profound influence on aboveground plant growth through intricate feedback mechanisms. Among soil nematodes, categories encompass bacterivores, fungivores, plant-parasitic, and omnivore–predator nematodes, each contributing to distinct carbon flow pathways, such as the bacterial channel, the fungal channel, and the root channel. Specifically, within these pathways, the bacterial channel involves the collaboration of bacteria and bacterivorous nematodes and the fungal channel involves fungi and fungivorous nematodes, while the root channels are inhabited by plant-parasitic nematodes (Thakur and Geisen, 2019). Increasing evidence shows that the interconnections between these channels play a critical role in regulating soil food webs (Ferris, 2010; Kou et al., 2020; Wu et al., 2021). The interaction between soil microorganisms and nematodes directly affects the connectivity of the soil micro-food web (Figure 1). This interaction holds significant implications for organic matter decomposition, carbon storage, nutrient recycling and redistribution, soil respiration, and aggregate formation (Ferris et al., 2001). Utilizing microorganisms and nematodes as a starting point for examining soil micro-food webs offers valuable insights into the nutrient cycling mechanisms in the ecosystem. It is of great scientific significance for the in-depth understanding of the underground ecological processes in which soil organisms participate under the addition of phosphorus and for revealing soil health and quality (Jiang et al., 2018).

Soil microorganisms and nematodes are pivotal contributors and indicators of subterranean ecosystems properly functioning. Microbial communities in the soil significantly influence carbon sequestration, nutrient cycling, decomposition of plant residue, and productivity supply (Fang et al., 2016). Various ecological indicators related to soil nematodes illuminate the response of the soil micro-food web to resources and their contribution to ecosystem functions (Ferris et al., 2001; Briar et al., 2012). Furthermore, the interactions between soil microorganisms and nematodes act as indicators of ecosystem health. Soil nematodes play a crucial role in shaping the composition of microbial communities. Alterations in the structure of the nematode community trigger dynamic shifts in the microbial community, impacting dominant species. This influence is evident in the selective predation of soil nematodes on specific microorganisms. In meeting their growth needs, soil nematodes may preferentially consume more rapidly growing microorganisms, leading to changes in the structure and diversity of microbial communities (Marschner and Kalbitz, 2003; Jiang et al., 2018; Wang et al., 2018). The bottom-up relationship between soil nematodes and microbial communities plays a pivotal role in regulating the structure of the soil ecosystem. This relationship is directly related to the soil biogeochemical processes, thereby promoting soil ecosystem function and altering the soil ecological environment (Sohlenius et al., 1988; Francini et al., 2018). In general, disturbances in the soil environment trigger the trophic chain through a bottom-up mechanism. This bottom-up regulation results in alterations within bacterial and fungal communities due to primary production or resource input, which subsequently extend to higher trophic levels (Kou et al., 2020; Nguyen et al., 2020). Environmental disturbance can lead to bottom-up regulation within the trophic levels of the soil micro-food web, potentially impacting critical ecosystem functions, including nutrient cycling and soil production (Thakur and Geisen, 2019). In a context where human activities exert multifaceted impacts on soil systems, the analysis of key factors that drive cascading effects within trophic interactions is vital for the effective management of soil health and plant productivity (Barnes et al., 2017).

To more accurately represent the pathways of the soil micro-food web and carbon utilization in nematode production and respiration, Ferris (2010) introduced the concept of the soil nematode metabolic footprint (NMF) as a measure of carbon fluxes within the soil nematode food web. Metabolic footprints can be divided into enrichment footprints and structural footprints. Enrichment footprints are the metabolic footprints of those nematodes that respond most quickly to resource enrichment. Structural footprints are metabolic footprints of nematodes at higher trophic levels that may have regulatory functions in the food web (Ferris, 2010). Scholars are increasingly recognizing the importance of soil nematodes as biological indicators of the impact of human and soil disturbance on soil food webs (Hu et al., 2016). Previous studies have shown that phosphorus addition mainly affects the quantity and quality of organic resources, thereby affecting the composition and activity of soil biological communities (Aerts et al., 2003). It has been suggested that low concentrations of phosphorus addition can improve soil nematode and bacterial community diversity, while too high phosphorus concentrations can improve soil fungal community diversity (Bissett et al., 2011; Hu J. et al., 2017). Consequently, we speculate that the outcomes of phosphorus addition will differ depending on the quantity applied. However, only some studies have attempted to explore the mechanisms by which soil food webs respond to such differences in resource management, and our study helps fill this knowledge gap.

The Loess Plateau in northwestern China has a long farming history. Due to the special ecological environment and soil texture, the Loess Plateau suffers from severe soil erosion, making it one of the most vulnerable regions in the world (Li et al., 2008; Fu et al., 2010). Alfalfa, an excellent legume forage for popularization and planting, has the characteristics of high yield, rich nutrition, and strong ecological adaptability. Its root system has strong nodule nitrogen (N) fixation, increasing soil organic matter, improving the regional ecological environment, and promoting the development of animal husbandry (Abbas et al., 2022). However, continuous alfalfa planting has seriously consumed soil moisture and phosphorus availability, resulting in a short, vigorous growth period and low alfalfa yield, and also caused the degradation of alfalfa artificial grassland (Gu et al., 2018; Wang et al., 2021). Nevertheless, the phosphorus addition exhibited a noticeable influence on the nitrogen fixation within alfalfa root nodules (Jia et al., 2006). Therefore, choosing the appropriate amount of phosphorus benefits the growth and soil fertility improvement of alfalfa. In summary, this study used an 8-year fertilization experiment to investigate the effects of different phosphorus addition on alfalfa soil and explore their effect on the decomposition pathway of soil micro-food web. We hypothesized that the amount of phosphorus application significantly influences the community structure, diversity, and metabolic footprint of microorganisms and nematodes, thereby impacting the decomposition pathways within the micro-food web. At low phosphorus concentrations, fungi, fungivorous nematodes, and predatory/omnivorous nematodes are primarily engaged in fungal decomposition pathways, enhancing carbon storage. Conversely, at high phosphorus concentrations, bacteria, bacterivorous nematodes, and predatory/omnivorous nematodes primarily participate in bacterial decomposition pathways, potentially reducing carbon storage.

## 2 Materials and methods

### 2.1 Site description

This study carefully selected an experimental site which is situated within the Gansu Agricultural University Comprehensive Experimental Station for dry farming on the Loess Plateau in the northwestern region of China. The experimental site belongs to Dingxi City in Gansu Province (35°28′N, 104°44′E with an average altitude of 1,970 m. In this area, the mean annual temperature is 6.4°C, the mean annual precipitation is 390 mm, and the mean annual evaporation is 1,531 mm. The experimental site is located in a semi-arid middle temperate zone area, a typical rainfed farming area with one crop annually. The basic properties of soil were characterized by organic matter content of 8.04 g kg^−1^, total nitrogen content of 0.82 g kg^−1^, and total phosphorus content of 1.07 g kg^−1^.

### 2.2 Experimental design and soil sampling

In this experiment, alfalfa was planted in April 2014 using a drill method with a seeding rate of 22.5 kg ha^−1^, 10 rows per plot, and a row spacing of 30 cm. It had four treatments, including no phosphorus (CK), low phosphorus with 60 kg ha^−1^ (P60), moderate phosphorus with 120 kg ha^−1^ (P120), and high phosphorus with 180 kg ha^−1^ (P180). Phosphorus fertilizer was applied every 3 years (2014, 2017, and 2020), and nitrogen fertilizer was applied yearly (N 50 kg ha^−1^). Each treatment with three replicates was applied to a plot with a dimension of 4 m × 3 m. The nitrogen fertilizer used in the experiment was urea (46% pure nitrogen), and the phosphorus fertilizer was superphosphate (12% pure P_2_O_5_). In addition to nitrogen and phosphorus fertilizers, no other organic and inorganic fertilizers were added in this experiment. The experiment was carried out under natural conditions without irrigation.

Soil samples were collected in June 2022 at the full flowering stage of alfalfa head stubble. There were 12 samples (4 treatments × 3 replicates). Soil samples were collected from the tillage layer of 0–20 cm depth. In each plot, five soil cores were randomly collected with a 2.5 cm diameter auger, and then, the samples were hand-mixed and passed through a 2-mm screen. Each sample was subdivided into two parts to determine general soil properties and stored at −80°C for the high-throughput sequencing of microorganisms.

### 2.3 Analysis of soil physicochemical properties

In this study, selected soil properties were measured, including soil moisture, pH, organic carbon (OC), total nitrogen (TN), total phosphorus (TP), and available phosphorus (AP). Soil water content (SW) was measured by drying fresh soil samples at 105°C to constant weight. Soil pH was measured in a soil: water (1:2.5) extract with a pH meter (Mettler Toledo, Switzerland). SOC was determined using the dichromate oxidation method (Sanmanee and Suwannaoin, 2009). This involved weighing 0.50 g of an air-dried soil sample, adding a potassium dichromate-sulfuric acid solution, and thoroughly mixing the sample and solution with a mixer, followed by sequential digestion and titration. Total nitrogen was analyzed by the Kjeldahl method (Brookes et al., 1985). First, 1.00 g of an air-dried soil sample should be weighed, fully enveloped in nitrogen-free weighing paper, and carefully positioned at the base of the digestion tube. Subsequently, 2 g of a copper sulfate-potassium sulfate accelerator and 5 mL of concentrated sulfuric acid are added to the tube, which was then covered with a curved neck funnel. The prepared sample was then placed in the digestion furnace and heated until the solution in the tube turns a light blue-green or off-white color. Upon completion of the digestion process, the tube was removed and allowed to cool to room temperature. Finally, the cooled sample was analyzed using a Kjeldahl Azotometer to determine its TN content. Total phosphorus was determined by the molybdenum blue method (Levine et al., 1955). Initially, 0.5 g of an air-dried soil sample was weighed in a digestion tube, which was moistened with a small amount of water. Next, 8 mL of concentrated sulfuric acid and 10 drops of perchloric acid were added. The tube was then placed in the digestion furnace for processing. Once digestion was complete, the tube was removed and allowed to cool to room temperature. The digestion liquid was then transferred from the tube to a 100 mL volumetric flask, diluted to the mark with water, and set aside for measurement. For the test solution, 10 mL was transferred to a 50 mL stoppered colorimetric tube, diluted with water to 40 mL, and supplemented with a drop of 2,4-dinitrophenol indicator. The pH of the solution was adjusted to a slight yellow hue using 0.5 mol/L sulfuric acid solution and 2 mol/L sodium hydroxide solution. Subsequently, 5 mL of molybdenum antimony antireagent was added, and the volume was brought up to 50 mL with water, followed by thorough mixing. After a 30-min interval, colorimetry was performed at a wavelength of 880 nm.

Soil AP was determined by the Olsen method (Do et al., 2007). Generally, 2.5 g of the air-dried soil sample was weighed and then transferred to a 250 mL conical flask. Then, 100 mL of sodium bicarbonate solution was added as the extraction agent, and the flask was securely sealed. Subsequently, the mixture was agitated on a constant-temperature reciprocating shaker for 30 min and then promptly filtered into a 150 mL dry Erlenmeyer flask using phosphorus-free filter paper, which was designated for analysis. Subsequently, 10 mL of the filtrate was transferred into a 50 mL volumetric flask, to which 5 mL of molybdenum antimony anti-chromogenic agent was added, and the volume was adjusted to the mark. After 30 min of resting period, colorimetric analysis was performed at a wavelength of 880 nm.

### 2.4 DNA extraction and high-throughput sequencing

The E.Z.N.A Soil kit (Omega Bio-tek, Norcross, GA, United States) isolated total genomic DNA from a 0.5 g soil sample, and the DNA concentration and purity were detected with a NanoDrop 2000 UV–VIS spectrophotometer (Thermo Scientific, Wilmington, United States). The quality of its extraction was detected by 1% agarose gel electrophoresis (Porazinska et al., 2009). The V4 region of the 18S rRNA gene of nematodes was amplified with the primers NF1(5′-GGTGGTGCATGGCCGTTCTTAGTT-3′) and 18S r2bR (5′-TACAAAGGGCAGGGACGTAAT-3′) (Beauregard et al., 2010). The PCR amplification was performed for each soil DNA extract in triplicate and combined into a single composite sample. The thermal cycling conditions were as follows: 95°C for 5 min, 35 cycles of 95°C for 30 s, 55°C for 30 s, and 72°C for 45 s, followed by 72°C for 10 min for primers NF1/18S r2bR. For bacteria, the V3-V4 region of the 16S rRNA gene was amplified using the primer pair 515F (5′-GTGCCAGCMGCCGCGG-3′) and 907R (5′-CCGTCAATTCMTTTRAGTTT-3′) (Wang and Wang, 1996). The thermal cycling conditions were as follows: pre-denaturation at 98°C for 2 min, denaturation at 98°C for 15 s, annealing at 55°C for 30 s, extension at 72°C for 30 s, and final extension at 72°C for 5 min, 30 cycles. For fungi, the ITS1 region was amplified using the primer pair ITS1F (5′-CTTGGTCATTTAGAGGAAGTAA-3′) and ITS1R (5′-GCTGCGTTCTTCATCGATGC-3′) (Yang et al., 2017). The thermal cycling conditions were as follows: 95°C for 5 min; 15 cycles of 95°C for 1 min, 50°C for 1 min, and 72°C for 1 min, followed by 72°C for 7 min for primers ITS1F/ITS1R. PCR products were gel-purified using the Wizard SV Gel and PCR Clean-Up System (Promega, San Luis Obispo, United States) (Mueller et al., 2014; Sinclair et al., 2015). The resultant PCR products were combined at equimolar concentrations before being sequenced on an Illumina MiSeq sequencer at the Majorbio Bio-Pharm Technology Co., Ltd. (Shanghai, China). Paired-end (PE) reads obtained from Illumina sequencing were initially spliced based on overlap relationships, simultaneously undergoing quality control and filtration. After distinguishing the samples, both OTU clustering and species taxonomy analyses were performed. Based on the OTU cluster analysis results, diversity index analysis was performed on OTUs. Using the taxonomic information, community structure statistical analysis was performed at the fungal and bacterial phylum levels and the nematode genus level. Based on the above analysis, in-depth statistical and visual analyses were conducted on community composition and phylogenetic information of each treatment. The microbial and nematode DNA sequences of the 12 soil samples were deposited in the SRA of the NCBI database under Accession nos. NCBI: PRJNA988166 and SRA: SUB13581533.

### 2.5 Data analysis

#### 2.5.1 Calculation of soil microbial and nematode diversity index

Calculate a diversity index of bacteria, fungi, and nematodes. The Chaol index reflected the richness of microbial and nematode communities (Chao, 1984), and the Shannon index indicated the diversity of microbial and nematode communities (Shannon, 1997).


$$\begin{array}{l} S_{\text{chao}1}=S_{\text{obs}}+\frac{n_{1}\left(n_{1}-1\right)}{2\left(n_{2}+1\right)}\\ \;H_{\text{shannon}}=-\sum_{i=1}^{s_{\text{obs}}}\frac{n_{i}}{N}\ln\frac{n_{i}}{N} \end{array}$$

where $S_{\text{obs}}$ is the number of OTUs observed; $n_{1}$ is the data with one sequence (e.g., singular); $n_{2}$ is the data with two sequences (e.g., even); $n_{i}$ is the number of OTUs containing sequence i; and N is the total number of sequences. The number of OTUs in the sample was evaluated using the chao1 index. The larger the Chao1 index, the larger the number of OTUs, indicating more species in the sample. The larger the Shannon value, the higher the community diversity.

Indices such as the nematode Basal Index (BI), Structural Index (SI), Enrichment Index (EI), Trophic Diversity Index (TD), Free-living Nematode Maturity Index (MI), Plant-parasitic Nematode Maturity Index (PPI), and Channel Index (CI) were served to reflect the food web structure, nutrient enrichment status, and decomposition pathways of soil ecosystems (Bongers, 1990; Ferris and Bongers, 2006).

#### 2.5.2 Metabolic footprints of nematode communities

The nematode metabolic footprints were calculated using the fresh weight of nematodes listed in the “Nematode-Plant Expert Information System.”^1^


$$NMF=\Sigma \;\left(N_{t}\times \left(0.1\times \left(W_{t}÷m_{t}\right)+0.273\left(W_{t}^{0.75}\right)\right)\right)$$

In the formula: $N_{t}$ is the abundance of t-type nematode group; $m_{t}$ is the c-*p* value of t-type nematode group; and $W_{t}$ is the biomass of t-type nematode group (Ferris, 2010).

Nematode flora analysis assessed enrichment footprints (Fe) and structural footprints (Fs). Taking the coordinate point of (SI, EI) as the center position, sequentially connecting (SI-0.5Fs/k, EI), (SI + 0.5Fs/k, EI), (SI, EI-0.5Fe/k), and (SI, EI + 0.5Fe/k), the formed diamond area was the functional metabolic footprint of the nematode community (Zhang et al., 2015).

#### 2.5.3 Energy flow analysis of soil food web

The energy flow analysis of the soil food web referred to the method by Ferris and Bongers (2006). The coordinate point of the vertex was (50, 86.6), the coordinate point of the lower left corner of the triangle was (0, 0), and the coordinate point of the lower right corner was (100, 0). Geometry knowledge calculated the coordinate points that determine the center point of a triangle and the midpoints of three sides. Seven coordinate points of each treatment were calculated from the relative metabolic footprints of bacterivorous, fungivorous, and plant-parasitic nematodes. After setting the coordinate points of each process, we selected all of them to draw a scatter diagram, in which the drawing of the three sides of the triangle and the midline of each side can be presented by adding a solid line or a dashed line, finally deleting the coordinate axis.

#### 2.5.4 Correlation ecological network and structural equation model

This study assessed the relationship between microorganisms and nematodes using correlation network analysis. To simplify the correlations between microbes and nematodes, we focused on the relative abundance at the genus level, thereby excluding interactions between genera within the same species. We screened for all possible Spearman correlations among genera appearing in at least three samples. If the Spearman correlation coefficient (*r*) > 0.6 and *p* < 0.05, there was a valid co-occurrence between genera (Deng et al., 2012). The network visualization was performed using Gephi v0.9.2.

Path analyses were used to reveal nematode metabolic footprints and microbial community relationships. The arrows and their path coefficients indicated the direction and strength of the relationships between these potential variables. Model fit to the data was evaluated using the X^2^ value and associated *p* value. Three tests, namely, Comparative Fit Index (CFI), Goodness of Fit (GFI), and Root Mean Square Error of Approximation (RMSEM), were used to assess model fit. The optimal model was identified by progressively eliminating non-significant paths. Path analyses were conducted using Amos 24.0 software (Arbuckle, 2006).

#### 2.5.5 Statistical analysis

One-way ANOVA was performed to investigate whether different phosphorus treatments significantly affected soil physicochemical properties, microorganisms, and nematode communities. Differences between all soil parameter means were compared using the least significant difference (LSD) (*p* < 0.05). Charts and figures of soil properties, microorganisms, and nematode communities were generated by SPSS Statistics 22 (SPSS Inc., Chicago, IL, United States) and Origin 2021, respectively. Canoco 5.0 software was used to determine the interactions between soil physicochemical properties, microorganisms, and nematode communities. The correlation network was carried out using R software packages “igraph.”

## 3 Results

### 3.1 Environmental factors

Soil physicochemical properties are shown in Table 1. There was no significant difference between SW and TN. SOC, TP, and AP increased significantly with increasing phosphorus addition (*p* < 0.05), while soil pH decreased with increasing phosphorus addition, and the P180 treatment was significantly higher than that of CK (*p* < 0.05).

**Table 1:** Soil physical and chemical properties under different phosphorus addition treatments.

Factors	CK	P60	P120	P180
Soil water (%)	7.75 ± 0.15a	8.04 ± 0.12a	7.69 ± 0.43a	7.89 ± 0.12a
Soil organic carbon (g·kg^−1^)	11.01 ± 0.12c	11.30 ± 0.07b	11.60 ± 0.06a	11.67 ± 0.03a
Total nitrogen (g·kg^−1^)	0.78 ± 0.01a	0.80 ± 0.02a	0.83 ± 0.01a	0.79 ± 0.01a
Total phosphorus (g·kg^−1^)	0.82 ± 0.01c	0.90 ± 0.01b	0.94 ± 0.02a	0.98 ± 0.01a
Available phosphorus (mg·kg^−1^)	4.07 ± 0.27c	5.57 ± 0.09b	6.05 ± 0.02a	6.21 ± 0.06a
pH	8.31 ± 0.02a	8.29 ± 0.01a	8.27 ± 0.02ab	8.24 ± 0.01b
C/N	14.13 ± 0.16a	14.18 ± 0.36a	13.98 ± 0.11a	14.69 ± 0.21a

Values are mean ± SE in triplicate replicates. Different lowercase letters indicate significant differences with a value of *p* < 0.05 based on the ANOVA. C/N, Ratio of carbon to nitrogen.

### 3.2 Community structure characteristics of soil microorganisms and nematodes

**Figure 2:**
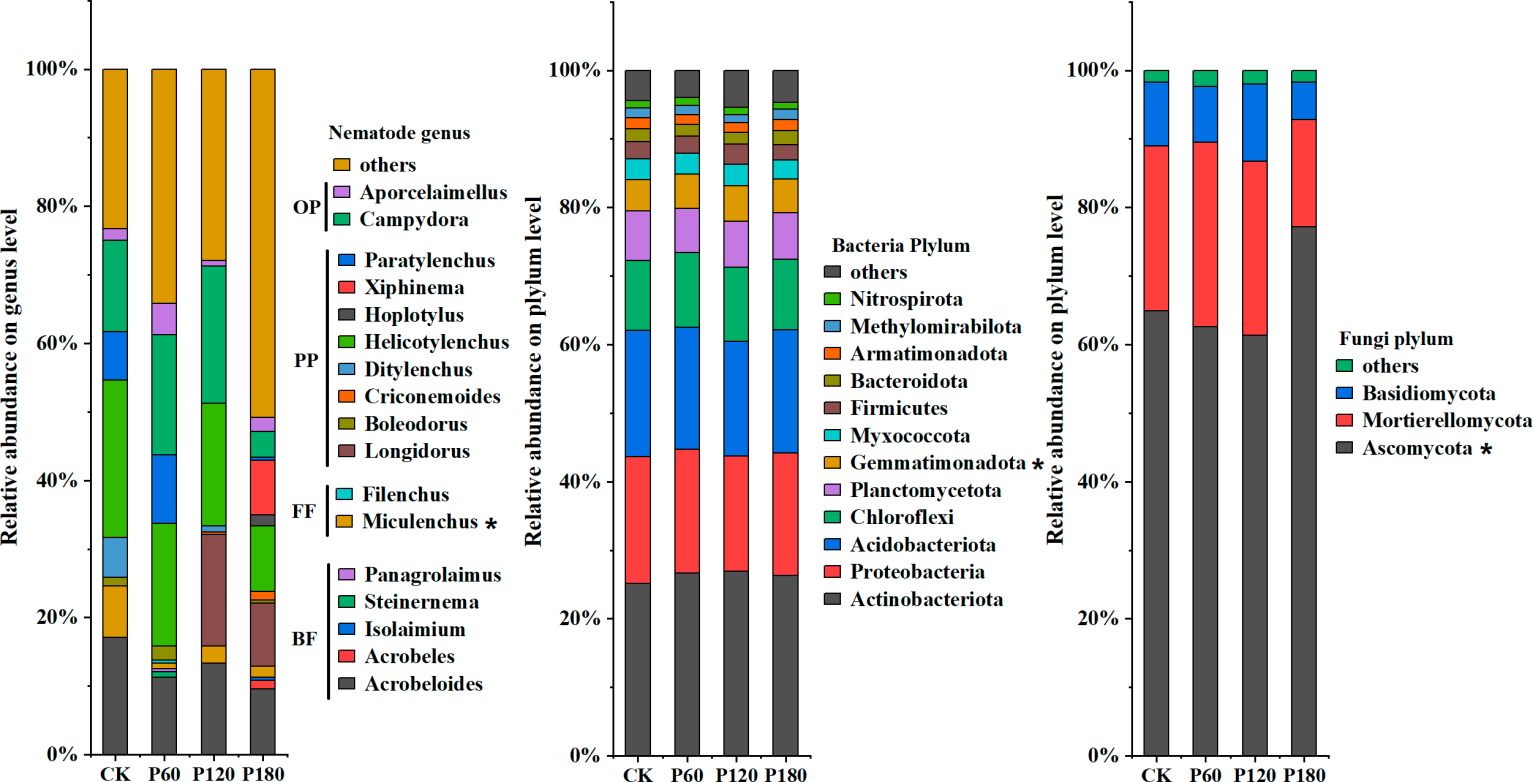
Relative abundance of soil microorganism and nematode communities (greater than 0.1%) in different phosphorus treatments: CK, P60, P120, and P180. Dominant genera: relative abundance of soil nematodes is greater than 10%; common genera: relative abundance of soil nematodes is 1–10%; rare genera: relative abundance of soil nematodes is less than 1%. BF, Bacterivorous nematodes; FF, Fungivorous nematodes; PP, Plant parasitic nematodes; and OP, Omnivorous predatory nematodes. The asterisk (^*^) indicated a significant difference between treatments (*p* < 0.05).

In this study, high-throughput sequencing was used to identify soil microorganisms and nematode communities under different phosphorus treatments (Figure 2). The results showed significant differences in the response of soil microbial and nematode communities to different phosphorus addition treatments (*p* < 0.05). At the soil nematode genus level, *Miculenchus* had significant differences between different phosphorus addition treatments (*p* < 0.05); at the bacterial phylum level, *Gemmatimonadota* showed a significant difference (*p* < 0.05), and at the fungal phylum level, *Ascomycota* displayed a significant difference (*p* < 0.05). In addition, different phosphorus addition treatments influenced the relative abundance of nematode trophic groups. Specifically, the relative abundance of plant-parasitic nematodes in CK and P120 treatments was significantly higher compared with P60 treatment and P180 treatment. The CK treatment demonstrated a significantly higher relative abundance of bacterivorous and fungivorous nematodes in comparison to other treatments. Conversely, the P180 treatment exhibited a noticeable decrease in the abundance of omnivorous-predatory nematodes compared with the other treatments.

### 3.3 Alpha diversity of soil microorganism and nematode communities

**Figure 3:**
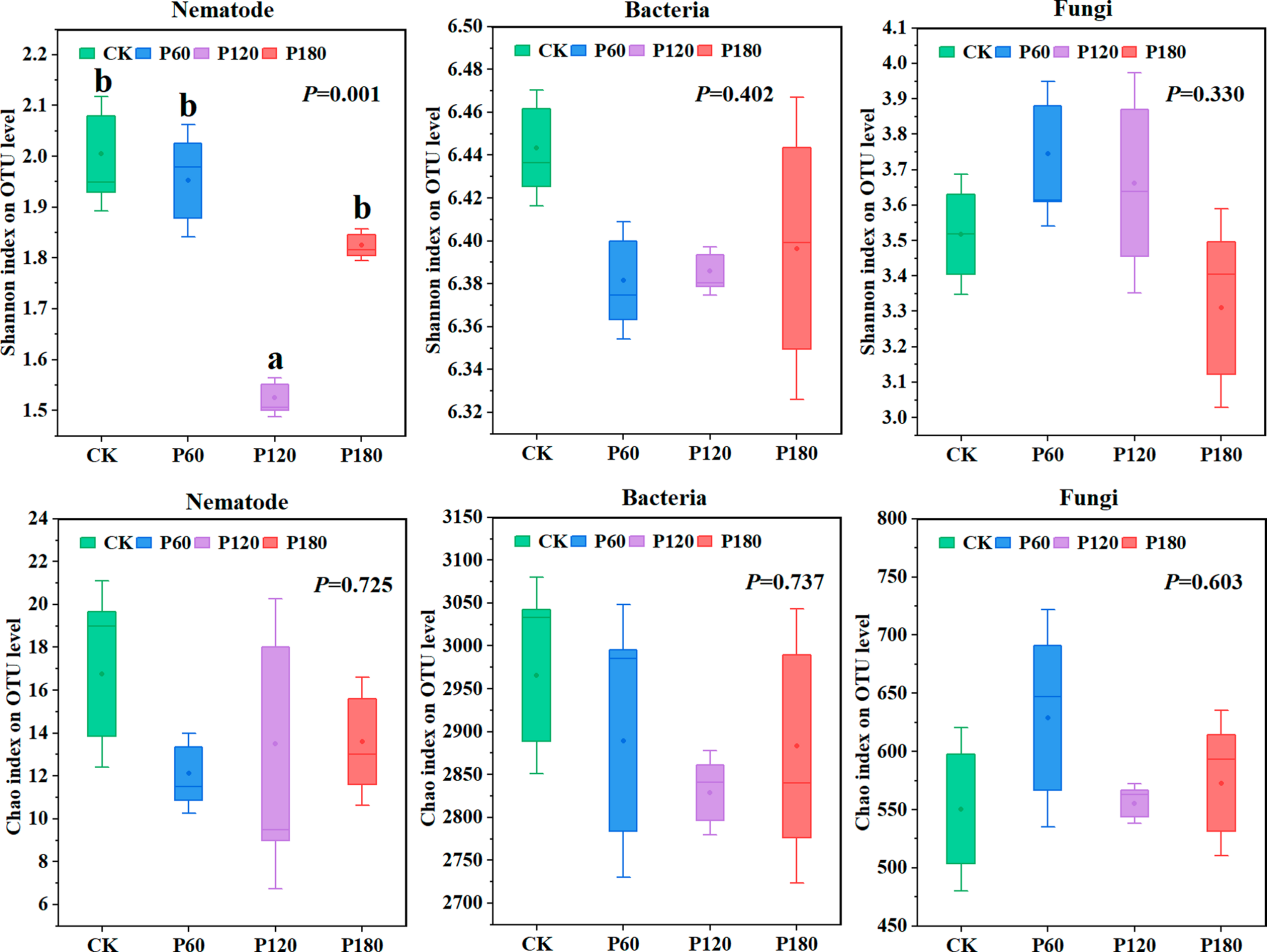
Diversity of soil microorganism and nematode communities under different phosphorus treatments. Different lowercase letters indicate significant differences with a value of *p* < 0.05 based on the ANOVA.

Shannon and Chao1 indexes of soil microorganisms and nematode communities (Figure 3) were measured to evaluate the α diversity of soil microorganisms and nematode communities under different phosphorus additions. Regarding the Shannon index, soil nematodes and fungi in the P60 treatment displayed a higher value than other treatments. Similarly, for the Chao1 index, soil bacteria and fungi under the P60 treatment surpassed other treatments.

### 3.4 Analysis of soil nematode metabolic footprint and food web energy flow

**Figure 4:**
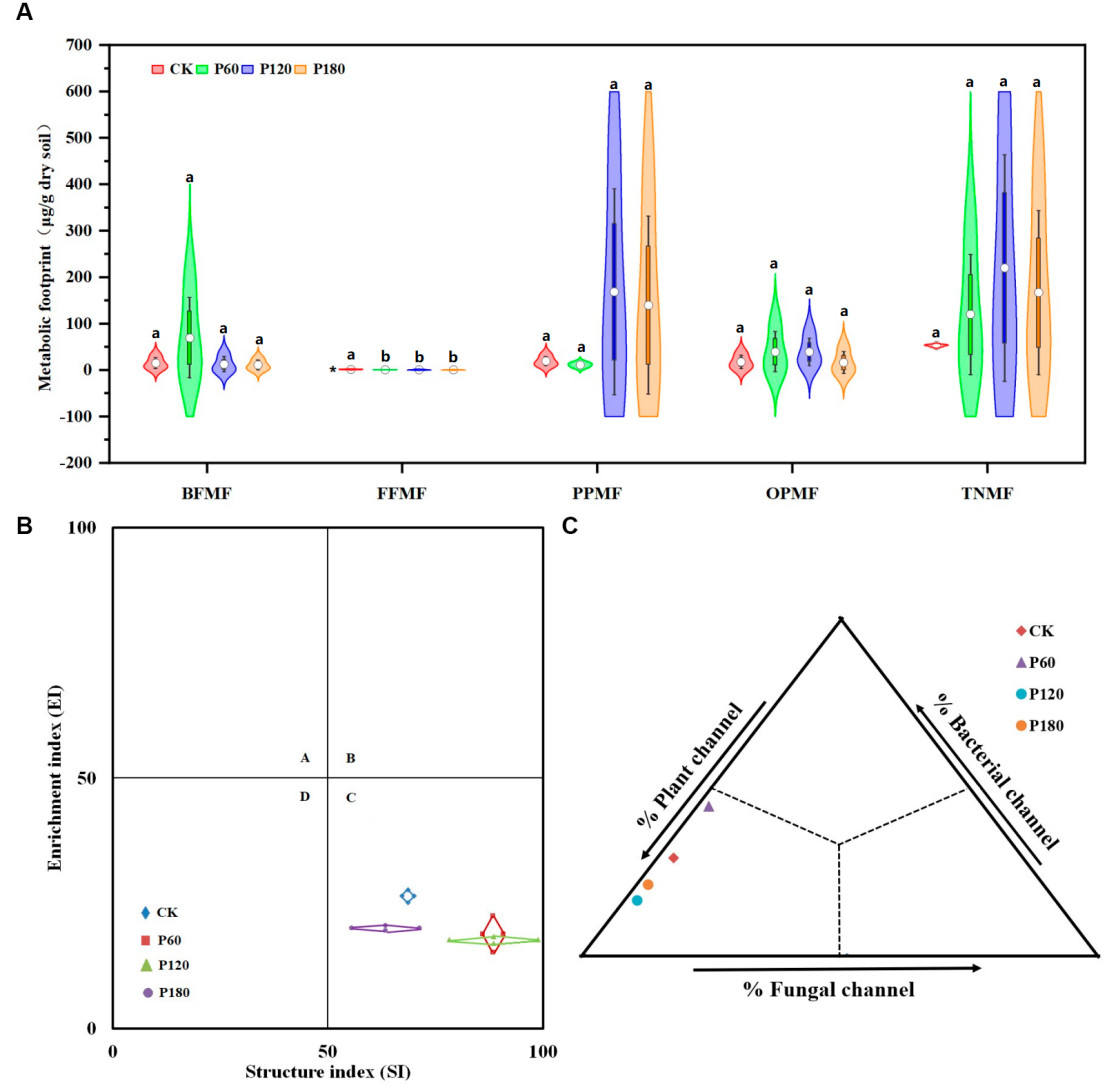
Metabolic footprint, floristic analysis, and food web energy flow analysis of soil nematodes. **(A)** Metabolic footprint; **(B)** Flora analysis; **(C)** Food web energy flow analysis; the piano box size depends on the interquartile range of the data. A large box means that the data distribution is discrete, and the data fluctuate greatly. Small means of the dataset are concentrated. The upper side of the box is the 75% quantile, the lower side is the 25% quantile, and the white dot on the violin plot represents the average value. BFMF, Bacterivores metabolic footprint; FFMF, Fungivores metabolic footprint; PPMF, Plant parasites metabolic footprint; OPMF, Omnivores-predators metabolic footprint; and TNMF, Total nematode metabolic footprint. The asterisk (^*^) indicated a significant difference between treatments (*p* < 0.05). Different lowercase letters indicate significant differences with a value of *p* < 0.05 based on the ANOVA.

The bacterivore’s metabolic footprint (BFMF) and omnivores-predators’ metabolic footprint (OPMF) in the P60 treatment were higher than those of other treatments. In contrast, the fungivore’s metabolic footprint (FFMF) and plant parasites’ metabolic footprint (PPMF) were lower than those of other treatments. The opposite was true for the P180 treatment (Figure 4A). Flora analysis showed (Figure 4B) that CK, P60, P120, and P180 treatments were all in the C quadrant. The functional footprint of soil nematodes (diamond area) shows that the P60 treatment is higher than other treatments. The soil nematode enrichment index (center position of the diamond) increased with the increased amount of phosphorus added. The soil nematode structure index (center position of the diamond) decreased with the increased amount of phosphorus added. Food web energy flow analysis (Figure 4C) showed that the P60 treatment had the largest proportion of bacterial and fungal energy flow channels. In contrast, the proportion of plant energy flow channels was the smallest, and the P120 treatment was on the contrary. P180 treatment was between P60 and P120 treatments, indicating that energy conversion and utilization efficiency in soil food webs at P60 treatment was relatively high.

### 3.5 Analysis of functional indices of soil nematode communities

**Figure 5:**
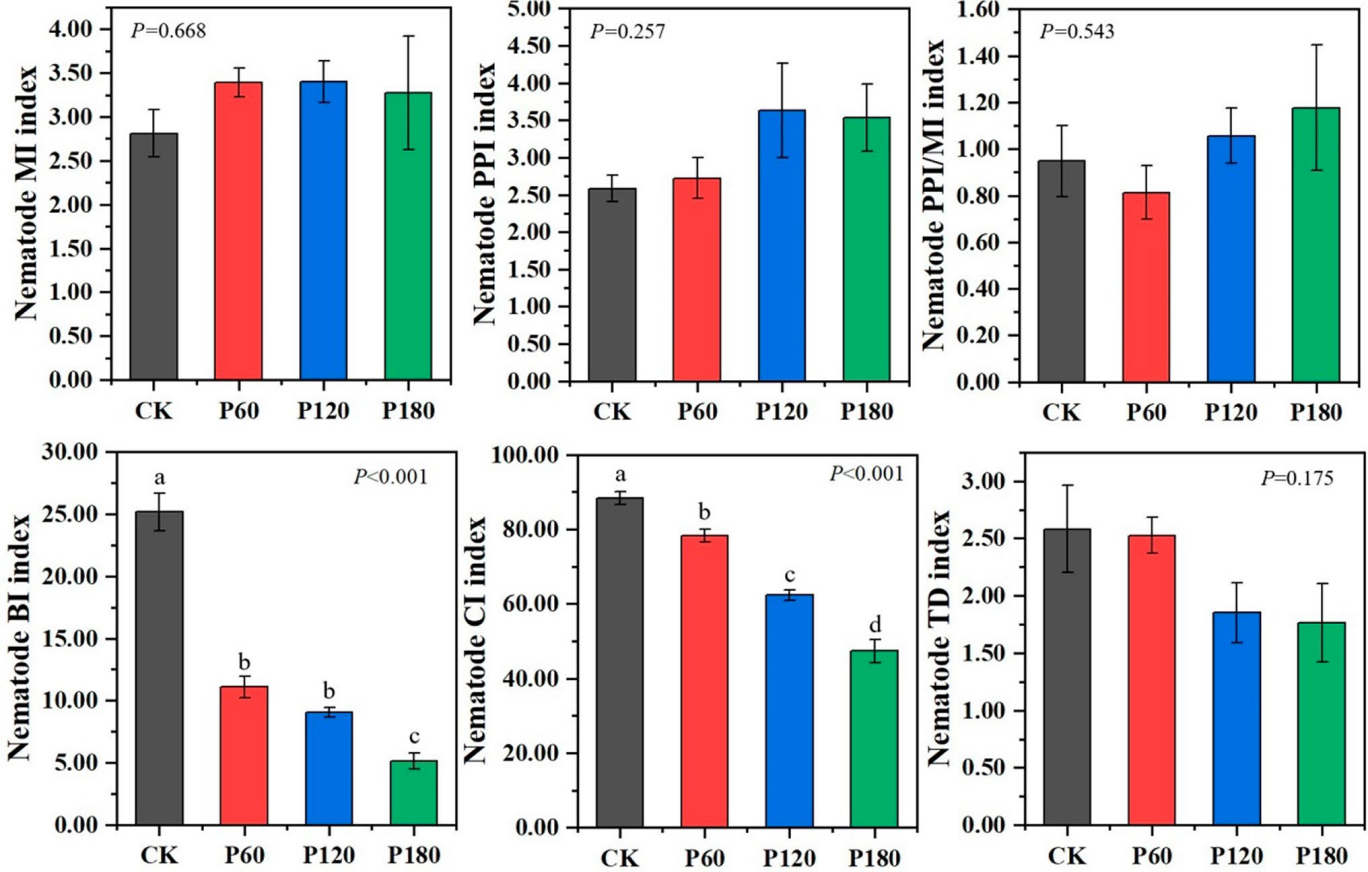
Functional structure index of soil microorganism and nematode communities under different phosphorus treatments. Different lowercase letters indicate significant differences with a value of *p* < 0.05 based on the ANOVA.

The functional index of the soil nematode community, as shown in Figure 5, reveals several findings. The Free-living Nematode Maturity Index (MI) for the P60 treatment surpasses other treatments. Conversely, both the plant parasitic nematode maturity index (PPI) and the ratio of PPI to MI (PPI/MI) were lower in the P60 treatment. These indexes suggested that the P60 treatment experiences fewer external disturbances, indicating a more stable ecological environment. As phosphorus application increases, indices such as the nematode basal index (BI), channel index (CI), and trophic diversity index (TD) demonstrate a declining trend. Significant differences (*p* < 0.05) were observed in BI and CI across treatments. This pattern indicated that lower phosphorus concentrations would reduce environmental disruptions, thereby enhancing the resistance of the soil food web. Furthermore, fungi predominantly dominated the degradation pathway in the soil food web at low phosphorus concentrations, while bacteria prevail at high phosphorus concentrations.

### 3.6 Soil micro-food web relationships

**Figure 6:**
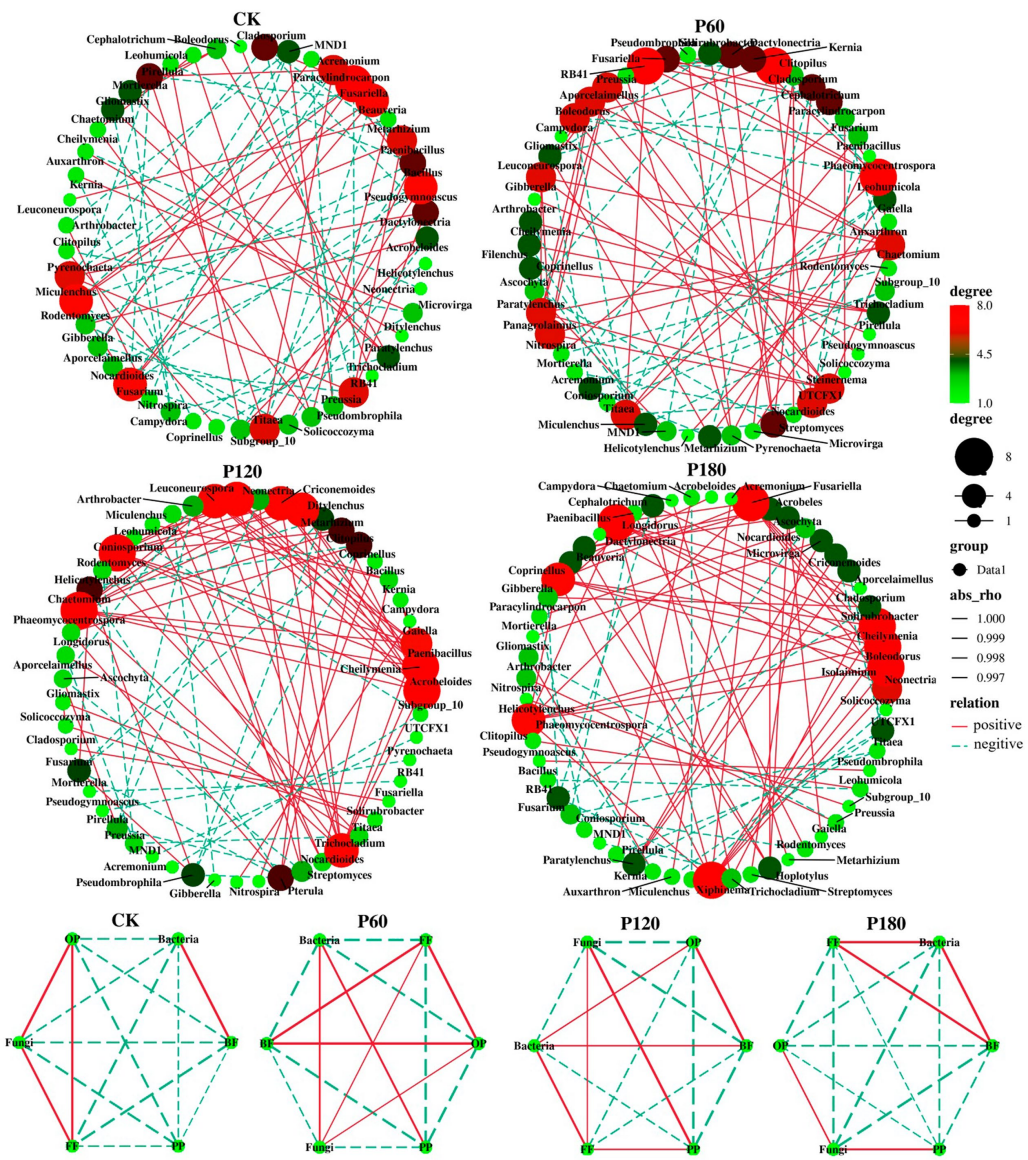
Network visualization of soil micro-food web interaction strength under different phosphorus treatments. The size of each node is proportional to the centrality score, with genera with larger centrality representing the key species of each network. Lines between each pair of nodes indicate a strong positive (red) or negative (dashed blue) interaction, and the thickness of the line indicates the strength of the correlation.

**Figure 7:**
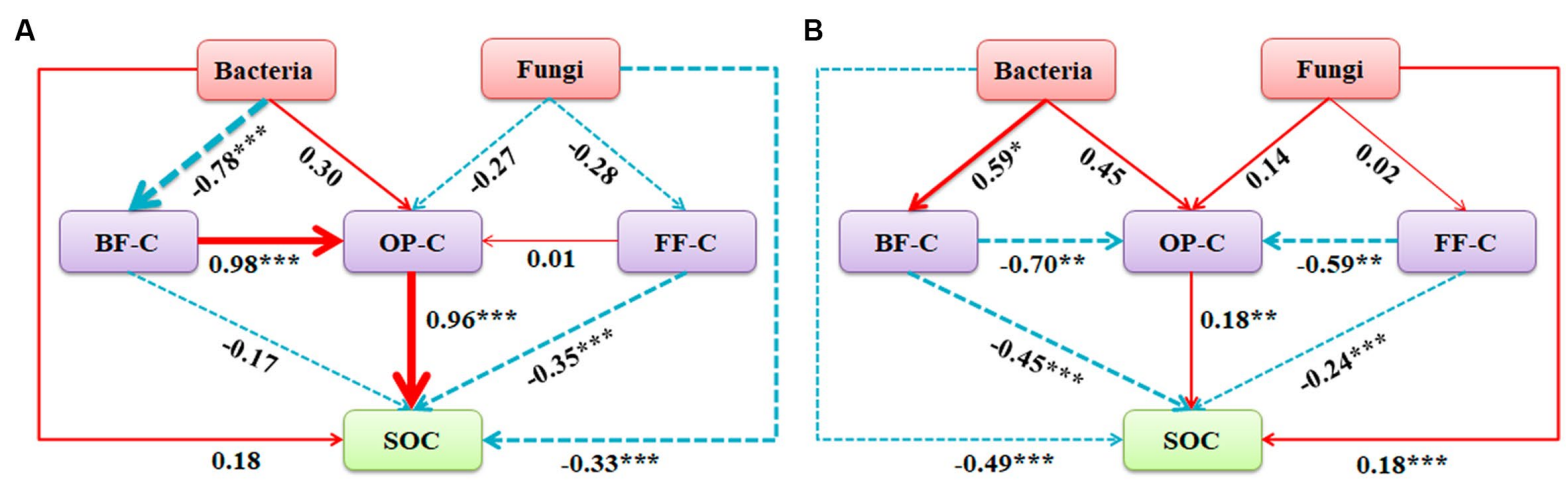
Path analysis model of decomposition pathway of soil micro-food webs with **(A)** low phosphorus concentration and **(B)** high phosphorus concentration. (**A**, X^2^ = 6.049, df = 3, *p* = 0.791, CFI = 0.865, GFI = 0.895, RMSEA = 0.078, NFI = 0.904, NNFI = 0.892; **B**, X^2^ = 3.445, df = 3, *p* = 0.328, CFI = 0.980, GFI = 0.906, RMSEA = 0.052, NFI = 0.924, NNFI = 0.903). The width of the arrow is proportional to the strength of the path coefficient, and the continuous red and broken blue arrows represent positive and negative relationships. Path analysis show direct and indirect effects of soil microorganism and nematode communities on carbon sequestration and bacterial and fungal community composition: first principal coordinate PCA1; BF-C, Carbon metabolic footprint of bacterivores; FF-C, Carbon metabolic footprint of fungivores; OP-C, Carbon metabolic footprint of omnivores-predators; SOC, Soil organic carbon.

The interaction between soil microorganisms and nematodes became less frequent and weaker as the level of phosphorus addition increased (Figure 6; Table 2). Compared with the CK treatment, the phosphorus addition treatment increased the total number of links in the network. Within the phosphorus addition treatment, the P60 treatment exhibited more links than other treatments, with increased negative connections and decreased positive connections. In contrast, the P180 treatment displayed an inverse trend. In the CK and P60 treatments, there was a positive correlation among fungi, FF, and OP. In the P120 treatment, a positive correlation existed between bacteria, BF, and OP. Meanwhile, the P180 treatment showed a negative correlation between OP and both BF and bacteria but a positive correlation between BF and bacteria. Consequently, the low phosphorus treatment revealed a positive correlation among fungi, FF, and OP, suggesting that fungi predominantly drive the soil food web degradation channel. In contrast, the high phosphorus treatment demonstrated a positive correlation among bacteria, BF, and OP, indicating bacteria as the primary degradation channel. Path analysis was utilized to evaluate carbon flow within the soil food web, taking into account diverse components of soil microorganisms and nematodes across varying phosphorus additions. The contribution rates of BF-C, FF-C, and OP-C to SOC were notably higher in the low-concentration phosphorus treatment compared with the high-concentration phosphorus treatment (Figure 7).

**Table 2:** Correlation network parameters under different phosphorus addition treatments.

Index	CK	P60	P120	P180
Number of nodes	49	52	51	57
Total links	84	101	103	92
Positive links	41	65	77	70
Negative links	43	36	25	22
Average degree	3.53	3.85	4	3.19
Average node size	8.62	9.07	8.33	8.13

### 3.7 Correlation analysis between soil microorganism and nematode communities and soil physicochemical properties

**Figure 8:**
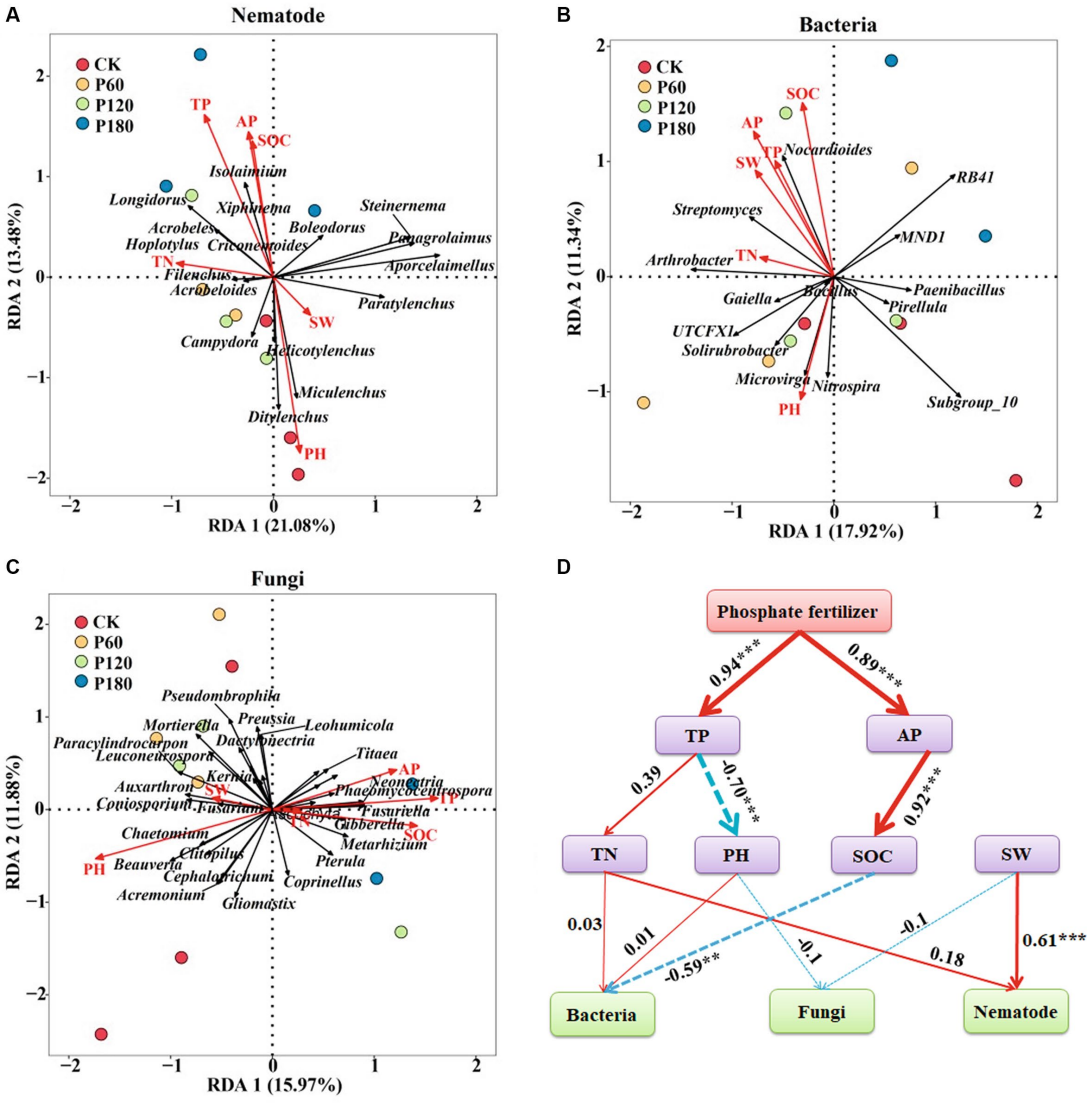
Correlation analysis between soil microorganism and nematode communities and soil physicochemical factors. **(A)** Redundancy analysis of soil nematode community and environmental factors. **(B)** Redundancy analysis of soil bacterial community and environmental factors. **(C)** Redundancy analysis of soil fungal community and environmental factors. **(D)** Path analysis model of soil microorganism and nematode communities and environmental factors (X^2^ = 72.43, df = 32, *p* = 0.791, CFI = 0.956, GFI = 0.921, RMSEA = 0.052, NFI = 0.944, NFI = 0.923). The width of the arrows is proportional to the strength of the path coefficient. The continuous red and broken blue arrows represent positive and negative relationships. Path analysis model show direct and indirect effects of environmental factors on microorganisms and nematode communities. SW, Soil water content; SOC, Soil organic carbon; TN, Total nitrogen; TP, Total phosphor; and AP, Available phosphorus.

The analysis employed soil microorganism and nematode community at the genus levels as response variables, with SW, pH, SOC, TN, AP, and TP serving as explanatory variables in the redundancy analysis (Figures 8A–C). Redundancy analysis of environmental factors and soil nematode communities showed that *Acrobeloides*, *Isolaimium*, *Filenchus*, *Hoplotylus*, and *Longidorus* were positively correlated with SOC, TP, AP, and TN; *Helicotylenchus*, *Ditylenchus*, and *Miculenchus* were positively correlated with SW and PH (Figure 8A). Redundancy analysis of environmental factors and soil bacteria communities showed that *Nocardioides*, *Streptomyces*, and *Arthrobacter* were positively correlated with SW, SOC, TN, AP, and TP; *Microvirga*, *Nitrospira*, *Solirubrobacter*, and *UTCFX1* were positively correlated with pH (Figure 8B). The effects of environmental factors on soil fungal communities showed that *Titaea*, *Neonectria*, *Phaeomycocentrospora*, and *Fusariella* were positively correlated with SOC, TN, AP, and TP; *Paracylindrocarpon*, *Coniosporium*, *Beauveria*, and *Chaetomium* were positively correlated with SW and PH (Figure 8C). The path analysis further indicated (Figure 8D) that SOC was the predominant environmental factor influencing the soil bacterial community (*p* < 0.05). In contrast, the soil nematode community was primarily affected by SW (*p* < 0.05). No single environmental factor significantly impacts soil fungal communities in isolation. Instead, soil fungal communities are influenced by a multitude of environmental factors. Both soil TP and AP exert indirect effects on microorganisms.

## 4 Discussion

### 4.1 Responses of microbial and nematode communities to physicochemical factors

P addition increased soil phosphorus availability, thereby affecting plant productivity and altering soil carbon inputs (Feng and Zhu, 2019). Moreover, phosphorus addition can change soil physical and chemical properties, thereby impacting the growth, activity, and community structure of the soil micro-food web components, ultimately affecting soil ecological functions. In this study, SOC emerged as the pivotal factor, impacting the bacterial community. As the main carbon source, SOC provided the energy and C-based nutrients, which are essential for soil bacteria. Bacteria can obtain more energy and nutrients when SOC levels are higher, which encourages bacterial growth and increases bacterial abundance. Simultaneously, changes in SOC concentration alter the interactions between soil microorganisms, including symbiosis, competition, and predation, all of which can have an impact on bacterial communities. Previous studies have shown that soil bacterial richness and abundance were significantly related to SOC content (Xia et al., 2011; Yuan et al., 2012), which was consistent with our research results. In terrestrial ecosystems, micro-food webs within soil ecosystems are instrumental in the carbon cycle, particularly in mediating litter decomposition and soil carbon sequestration. The process of soil carbon sequestration is influenced by an array of factors, including soil microbial biomass, community structure, secondary metabolites, and soil physical and chemical characteristics (Frey et al., 2006). Furthermore, the origin of stable soil carbon is predominantly driven by microbial activities, underscoring the pivotal role of microorganisms in stable carbon synthesis. Notably, the contribution to soil carbon sequestration varies between bacteria and fungi. Studies indicated that communities dominated by fungi have a superior capacity for carbon sequestration compared with those dominated by bacteria (Frey et al., 2006; Grandy and Neff, 2008). Soil nematodes predominantly inhabited the water film surrounding soil particles. Their vital activities, including movement, predation, and reproduction, are intricately linked to soil moisture levels (Yeates, 1979). Enhanced soil moisture facilitates these activities, resulting in a rise in both the abundance and diversity of nematodes. Additionally, increased moisture content improves resource availability, which indirectly influences nematode community dynamics. This suggests that soil nematode communities are influenced not only by direct environmental factors but also by indirect bottom-up effects stemming from substrate availability (Hu Z. et al., 2017). Redundancy analysis revealed a close relationship between soil pH and the communities of soil bacteria, fungi, and nematodes. Numerous studies have identified soil pH as the primary determinant in shaping microbial communities (Fierer and Jackson, 2006; Lauber et al., 2009), which is consistent with the results of this study. Microorganisms exhibit varying pH preferences and tolerances. Long-term phosphorus addition has been shown to lower soil pH, subsequently altering microbial community composition and diversity. This shift in pH is beneficial to certain microbes in new environmental conditions while suppressing or diminishing others. Additionally, soil pH influences nutrient forms and availability, subsequently impacting microbial growth and activity. Alterations in microbial communities consequently affect the composition and diversity of microbivorous nematodes, as these nematodes depend on microorganisms for sustenance. Changes in microbial populations directly influence their food sources and habitats. Thus, soil pH exerts a bottom-up effect on the entire microbial food web, initiating microbial community alterations and extending to higher trophic levels.

### 4.2 Effects of phosphorus addition on structure and function of soil micro-food web

Rhizosphere nutrients constitute the fundamental energy source of the soil micro-food web. Plant roots and their secretions significantly influence the energy flow within the soil micro-food web, stimulating the proliferation of plant-parasitic, bacterivorous, and fungivorous nematodes. This proliferation, in turn, impacts the micro-food web through the predation of these nematode types by higher trophic-level nematodes (Yeates and Bongers, 1999). Our study indicated that high phosphorus concentrations were advantageous for fungivorous and plant-parasitic nematodes (Porazinska et al., 2009). This may be because high concentrations of phosphorus led to an increase in soil fungal populations, the primary food source for fungivorous nematodes, resulting in a higher proportion of fungivorous nematodes (Beauregard et al., 2010). Moreover, phosphorus addition increased soil nutrients, stimulated alfalfa root growth, and promoted the reproduction of herbivorous nematodes, thereby increasing the proportion of plant-parasitic nematodes (Beauregard et al., 2010; Xiaofang and Li, 2020). This finding confirmed the strong interplay between plant roots and plant-parasitic nematodes and supported the previous research to a certain extent. Microbivorous nematodes not only expedite micro-food web turnover by feeding on microorganisms but their metabolic processes also provide feedback effects on plant growth. The interaction between soil nematodes and microorganisms transcends a simple predator–prey dynamic. Different nutritional types of nematodes exert varied impacts on microbial communities. Research has shown that soil nematode activity significantly inhibited bacterial and fungal proliferation. Furthermore, the existence of microbivorous nematodes increased the complexity and variability of the soil ecosystem and micro-food web structure (Sauvadet et al., 2016). The results of this study showed that fungi in low-concentration phosphorus soils have a negative correlation with plant-parasitic nematodes and a positive correlation with fungivorous nematodes and omnivorous-predatory nematodes. In high-P soils, bacteria exhibit a negative correlation with plant-parasitic nematodes and a positive correlation with bacterivorous and omnivorous-predatory nematodes. Analysis based on the structural characteristics and energy flow direction of the soil micro-food web suggested that a short-term increase in plant-parasitic nematodes elevated the consumption of plant root nutrients. Because nematodes were larger and had longer survival times, their community dynamics lag behind those of microorganisms. Simultaneously, in environments with different phosphorus concentrations, bacteria and fungi are more susceptible to external soil stress, affecting community growth, thereby regulating soil nematode communities through a bottom-up approach.

Energy flow pathways in the soil food web, classified based on energy sources, comprise bacterial, fungal, and plant-based channels (Moore and William Hunt, 1988). The varied feeding habits of nematode trophic groups enable them to represent these energy flows at a higher trophic level (Deng et al., 2012). Analysis of these flows revealed that in P60 treatment soil, bacterial and fungal pathways predominate, while the plant energy flow channels had the smallest proportion. This dominance suggests a relatively high efficiency in energy conversion and utilization within the P60 treatment soil food web. Specifically, the P60 treatment soil nematode community exhibited an elevated carbon utilization rate and an enhanced capacity to regulate the food web, maintaining a balanced predator–prey dynamic (Deng et al., 2012). Our research indicated that both the biomass of fungivorous nematodes and the total nematode biomass are greater in low-P soils compared with high-P soils. This disparity suggests that low-P soils, being less disturbed, offer more stable conditions for nematodes, particularly for large-sized k-strategy species treatment (Ferris, 2010). Fauna analysis demonstrated that the functional footprint of soil nematodes in the P60 treatment exceeded as compared with other treatments, implying that the P60 treatment enhanced soil nutrient levels and improved the soil environment. Consequently, this results in reduced soil disturbance and a more mature, stable food web structure. Furthermore, nematode functional metabolic footprints were larger at lower phosphorus concentrations, indicating a greater carbon flux through the decomposition pathway of the soil food web. This increase results in a greater allocation of carbon to higher trophic levels, where it was fixed by organisms at these levels (Ferris and Bongers, 2006).

The free-living nematode maturity index (MI) and the plant-parasitic nematode maturity index (PPI) were served as key indicators of high-intensity interference in a short period (Lenz and Eisenbeis, 2000). A higher MI value suggests a less disturbed environment and a more stable nematode community, while the opposite was true for a more intense disturbance (Neher, 2010). The plant PPI represents the proportion of plant parasitic nematodes selected by r2 and k2, which can reflect the ability of such nematodes to resist habitat disturbance and their reproductive strength (Ferris et al., 2001). Compared with MI and PPI, the ratio of maturity index of plant parasitic nematodes to free-living nematodes (PPI/MI) played an important role in responding to the soil ecological environment and coping with external environmental interference. It was more representative in terms of the subsequent recovery status. The higher PPI/MI value indicated a greater degree of disturbance in the habitat where the nematode community resided (Bongers, 1990). In this study, applying low-concentration phosphorus fertilizer improved the MI and reduced PPI and PPI/MI. These results suggested that low phosphorus levels enhanced soil environmental stability. Such an outcome arises because prolonged phosphorus addition compels local organisms to develop survival mechanisms that counter the effects of low-concentration phosphorus fertilizers. Consequently, the P60 treatment displayed the highest MI value and the lowest PPI and PPI/MI values, indicating minimal external disturbances and a more stable ecological environment. In contrast, the MI values diminished in the P120 and P180 treatments while the PPI and PPI/MI values increased. This trend may be attributed to the enhanced soil nutrients from phosphorus addition, promoting robust growth in above-ground plants and fostering healthy root systems. Such conditions deter plant-parasitic nematodes, demonstrating that high phosphorus concentrations reduce plant-parasitic nematode viability and hindering their survival and reproduction. Overall, this research supported the use of phosphorus fertilizers in restoring degraded grasslands.

### 4.3 The effect of phosphorus addition on the decomposition pathway of soil micro-food web

The ecological network indicated a positive correlation among fungi, FF, and OP at low phosphorus levels but no positive correlation between bacteria and BF. This implies that, under low phosphorus addition, the decomposition pathway of fungi exhibited greater strength compared with high phosphorus levels. Bacteria and fungi employ distinct metabolic abilities for the breakdown of substrates with varying qualities. The alteration in decomposition pathways within the soil food web played a crucial role in influencing the rate of soil carbon loss. Notably, the bacterial decomposition pathway, conducive to rapid nutrient turnover, surpassed the fungal decomposition pathway in this aspect (de Vries et al., 2011). The quality of readily available refractory substrates affects the transition of bacterial and fungal decomposition pathways in soil food webs, thereby largely influencing carbon turnover (Fabian et al., 2017). In this study, elevated phosphorus addition levels resulted in the predominance of bacterial decomposition pathways within the soil food web. However, as phosphorus concentration diminishes, the substrate exhibits increased resistance to degradation, thus favoring fungal decomposition. Therefore, increasing phosphorus may drive the successional trend of the community from fungi to bacteria. In addition, it was also observed that organic carbon increased at low phosphorus supply and relatively strong fungal decomposition pathways, suggesting that fungi had larger protective carbon pools and larger carbon retention ratios than bacteria. This effect improved the physical environment for carbon stabilization and promoted the accumulation of microbial-derived organic matter (Frey et al., 2006; Danger et al., 2016). Variations in both bacterivores and fungivore nematodes were driven by phosphorus supply and soil microbial communities (Ferris and Matute, 2003). Under different phosphorus levels, this study found that fungi, fungivores, and omnivores-predators were positively correlated at low phosphorus supply. However, bacteria, bacterivores, and omnivores-predators were positively correlated at high phosphorus supply, positively supporting the view that low-P and high-P supply enhance fungal and bacterial decomposition pathways, respectively (Rooney et al., 2006). Moreover, the decomposition pathways of bacteria and fungi in the soil food web need to be separated, as the two pathways work simultaneously, and different carbon transfer and utilization processes occur between them (Prescott and Vesterdal, 2021). Different phosphorus supplies regulated the community composition and decomposition pathways of microorganisms and nematodes differently.

Basis index (BI), channel index (CI), enrichment index (EI), and structure index (SI) can provide a large amount of information for food web dynamic processes in environments of stress, enrichment, stable structure, and rapid decomposition (Ferris and Tuomisto, 2015). Compared with other treatments, the enrichment index (EI) under the P60 treatment decreased, indicating that the number of Ba1 and Fu2 nematodes, such as *Acrobeles*, *Acrobloides*, and *Miculenchus*, was significantly reduced. A lower enrichment index (EI) value indicated a decrease in food resources at this level of phosphorus addition (Bongers et al., 1997), leading to fewer nutrient inputs from external sources into the soil. The elevated structural index (SI) under P60 treatment was attributed to the elevated number of cp3-5 nematode groups, especially the omnivore-predator cp3-5 nematodes, which may signify a higher degree of connectivity in the soil food web, with longer food chains, greater soil resilience (Ferris et al., 2001), and a more stable soil food web. The CI values were greater than 60 under the CK, P60, and P120 treatments and less than 60 under the P180 treatment. This distinction confirmed that soil food webs with lower phosphorus concentrations predominantly decompose via fungal channels, while those with higher phosphorus concentrations used bacterial channels. The decomposition of organic matter in the soil food web was carried out through different channels. Fungi break down substances that are rich in cellulose, lignin, and high carbon-to-nitrogen ratios, whereas bacteria process nitrogen-rich substances (Yeates et al., 1993; Wardle et al., 2003). Excessively higher nitrogen content also raised carbon content. However, a high soil organic matter C/N ratio can be crucial for long-term sustainable plant production in perennial or natural systems. In this state, the degradation pathway in the soil micro-food web is dominated by fungi (Ferris et al., 2001). As the phosphorus fertilizer concentration increases, the BI index gradually decreases. This decline suggested that greater phosphorus addition might amplify environmental disturbances, thus undermining the resilience of the soil food web. This may be related to the potential toxic impact of phosphorus addition on soil nematodes (Ferris and Bongers, 2006). Indeed, phosphorus supply serves many purposes and benefits. In addition to carbon fixation, phosphorus is a component of many important organic compounds that constitute crops. Phosphorus is present in nucleic acids, nucleoproteins, phospholipids, phytochemicals, adenosine phosphate, and many enzymes in crops. It plays an important role in energy conversion and metabolism of crops, affecting the synthesis and operation of carbohydrates and nitrogen and fat metabolism. It also enhanced the resistance of plants against stresses, such as drought, cold, and salinity.

## 5 Conclusion

This study has enhanced our comprehension of the mechanisms, governing alterations in soil micro-food web decomposition pathways under varying phosphorus supply conditions. Within the decomposition pathway of the soil micro-food web, phosphorus supply facilitated a noticeable shift between bacteria and fungi, thereby promoting the transition between bacterivores and fungivores. Notably, our experimental findings elucidate that low phosphorus supply reinforces the fungal decomposition pathway, whereas high phosphorus supply strengthens the bacterial decomposition pathway. The intricate nutrient interactions between soil microorganisms and nematode communities emerge as primary drivers of carbon flow in the decomposition pathways. Consequently, low phosphorus supply exerts a more pronounced regulatory effect on the decomposition pathway of the soil food web compared with high phosphorus supply, impacting the metabolic processes of carbon within the soil food web.

## Data availability statement

The datasets presented in this study can be found in online repositories. The names of the repository/repositories and accession number(s) can be found in the article/supplementary material.

## Author contributions

LiaL: Data curation, Investigation, Methodology, Software, Writing – original draft. ZL: Conceptualization, Funding acquisition, Methodology, Writing – review & editing. LinL: Conceptualization, Data curation, Methodology, Writing – review & editing. YN: Conceptualization, Investigation, Methodology, Writing – review & editing. YZ: Investigation, Methodology, Software, Data curation, Writing – review & editing. RH: Investigation, Project administration, Software, Supervision, Writing – review & editing. JL: Investigation, Methodology, Software, Writing – review & editing. LN: Data curation, Formal analysis, Investigation, Software, Supervision, Writing – original draft.

## Footnotes

^1^ http://plpnemweb.ucdavis.edu/nemaplex
